# Service quality, satisfaction and intention to use Union Digital Center in Bangladesh: The moderating effect of citizen participation

**DOI:** 10.1371/journal.pone.0244609

**Published:** 2020-12-28

**Authors:** Bikram Biswas, Sajib Kumar Roy

**Affiliations:** 1 Department of Educational Administration, Noakhali Science and Technology University, Noakhali, Bangladesh; 2 Department of Development Studies, Hajee Mohammad Danesh Science and Technology University, Dinajpur, Bangladesh; Univerza v Mariboru, SLOVENIA

## Abstract

This paper examines the service quality, satisfaction and intention to use Union Digital Center (UDC) in Bangladesh: The moderating effect of citizen participation. The study intends to measure the quality of service on the basis of satisfaction by adopting citizen participation as a moderator. Theoretically this study has used DeLone & McLean Information Systems (D&M IS) Success Model. The existing studies of Bangladesh are mostly qualitative and the correlation between the quality of service, satisfaction, and desire for using UDC has not verified. This research has adopted the D&M IS model while measuring and verifying the service quality based on satisfaction and use intention. A structured questionnaire method was used and data collected from 499 respondents from 10 UDC of 10 upazila under 8 divisions in Bangladesh. Partial Least Square (PLS), a statistical method that emerged on the basis of Structure Equation Modeling (SEM), technique has been used while analyzing the data. The result of this study has showed the quality (p<0.05) of information, system and service of UDC affects citizen satisfaction effectively where the moderator of citizen participation is also significant. This paper has constructed on the basis of a model and empirical data to verify the moderating effects of citizen participation. To ensure the improvement of service quality of UDC all of the dimensions related to the quality of service should be modified, develop the administrative system and citizen should be encouraging to participate in all aspects of services.

## 1. Introduction

Nowadays, Information and Communication Technologies (ICTs) are accepted as a powerful tool for the development of the socio-economic condition. Bangladesh started implementing Union Information and Service centers (UISCs) from 2010 for bringing public services to the door-step of grassroots and establishing transparency and accountability to them. The UISCs were renamed as Union Digital Centers (UDCs) in 2014. Through public-private-partnership, UDCs were expanded in 4554 unions around the country as a one-stop service center. UDCs delivered 45 million services to rural people during 2013–2015 [1] UDC is a useful and vital initiative to implement Digital Bangladesh Vision-2021 successfully. The functioning of UDCs is being operated by local entrepreneurs, i.e. one male and female at the premises of Union Parishad (UP), the lowest tier of local government by which the government connects itself with millions of people in terms of ensuring citizen’s access in public services. The nature of the work of UDC saves time and cost of users and service providers. So, UDC has been playing an active role in terms of achieving the goal of Digital Bangladesh.

For improving the quality of the public life and the advancement of the country, there is no alternative way of providing e-services timely to the people of all segments of society on an equal footing. By using digital platforms and modern communication facilities, the government’s service provision mechanism is becoming more responsive to the citizens’ needs (Zaman 2015). The establishment of UDC has opened up the gateway to the rural people according to getting ICT facilities that were not available to them. Now the question is, how does the SQ measure in the process of construction and operation of UDCs? How do we know that the public is satisfied or not? What’s the role of citizen participation in these issues? These are all issues that need to be understood and resolved.

## 2. Literature review

Compare with globally, a few numbers of studies have been done on UDC authorized model to provide e-services to the rural people of Bangladesh. Most of the service receivers of the rural areas need to meet officials of these public offices for receiving available services and information such as welfare benefits, education, health, agriculture, and information to know about the market prices [2]. Chatfield and Al Anazi (2013) developed a model of citizen trustworthiness with different delivery options of the government’s e-service [3]. After empirical analysis on more than 400 data from the Saudi citizens who use transactional services of e-government, it was found that quality of service and citizen satisfaction describes the loyalty of citizens with the facilities of e-government. Suphattanakul (2014) examined the impact of public participation of local government’s SQ and how organizational culture moderates the impact of the public participation on the perception of the quality of service from the perspective of Thailand’s local government [4]. Breitenbach (2013) illustrated that how telecenters of Africa had played a vital role in the ICT arena for the development of the economic condition, education, health, etc. in the study namely "Telecenters for Sustainable Rural Development: Review and Case Study of South African Rural Telecenters." This paper also has shown that how telecenters had developed rural people’s lives of Thabina in Africa [5]. Mahmood (2005) identified the challenges and opportunities of Multipurpose Community Telecenters’ (MCTs) establishment in the rural areas of Pakistan, where authors focused on the purpose of MTC’s establishment regarding the formulation of policy, management, planning, technology, service, sustainability, etc. [6].

### 2.1. Service quality in the public sector

The quality of service has been recognized as an essential aspect to measure the public sector’s performance since the 1990s. In the IS literature, information quality (IQ) indicates the information produced and provided by the system [7]. Similarly, in the field of e-government, the quality of data is necessary for the government to make this information available on its website for the people [8]. System quality also focuses on the actual system, which can produce the output [7]. Service quality (SQ) has been defined in this study as a combination of the characteristics of system quality, IQ, and customer satisfaction. Kumar et al. (2007) claimed the relevance of studying the correlation between customer satisfaction and SQ but did not test it empirically [9]. Wangpipatwong et al. (2009) also mentioned the relevance of the correlation between SQ and citizen satisfaction based on Thailand's e-government [10]. SQ is the difference between the expectations of the customer regarding service and the perceptions of received service [11].

### 2.2. Citizen satisfaction

The term satisfaction was initially defined by Locke from the perspective job performance. It was described as “a pleasurable or positive emotional state resulting from the appraisal of one’s job” [12]. Oliver (1981) also explained satisfaction as "the summary of the psychological state resulting when the emotion surrounding disconfirmed expectations is coupled with the consumer’s prior feelings about the consumption experience" from the view of consumption context [13]. Research conducted by Olever (1997) on customer satisfaction mentioned that disgruntled clients are not willing to pose loyalty to the services or products they dislike [14].

### 2.3. Citizen participation

Citizen participation in the process of applying and receiving services that should be a kind of positive interaction. It is closer to “customer engagement” in the marketing field through self-service or cooperation with service personnel where customers actively participate and helps to create service value [15] thus reducing costs, and get psychological satisfaction [16]. In this research, citizen participation can be defined as interaction and cooperation with service providers through relevant information, materials, time, and other resources that will help to assist the UDCs in providing complementary services and create appropriate service values.

### 2.4. The moderating effects of citizen participation

The concept of "citizen participation" originated before and after the World War II. It was use of civil power and redistribution of power so that people who cannot control power in political, economic, and other activities now they have their own opinions. In short, citizens are trying to influence all of public policy and public life activities where they use information science and technology to develop their forms. As far as the specific research environment of this research is concerned in the Union Digital Centers (UDC). Citizen Participation is the process of applying and receiving services that should be a kind of positive interaction. Its main purpose is to affecting public policies and public life rather than obtain services related to itself. Therefore, it is closer to “customer engagement” in the marketing field through self-service or cooperation with service personnel where customers actively participate and also helps to create service value [15], thus reducing costs, and get psychological satisfaction [16], In this research, citizen participation can be defined as: Interaction and cooperation with service providers through relevant information, materials, necessary time and other resources that will help to assist the Union Digital Centers to provide corresponding services and to create relevant service values.

## 3. Hypothesis and theoretical model

In the area of IT SQ research, there are two research models that are widely used. One is the SERVQUAL SQ model proposed and revised by Parasuraman, Zeithaml, and Berry [11]. D&M IS success model is another one proposed and revised by DeLone and McLean. In this model, efficiency and operational quality of the IS are comprehensively evaluated by the three-dimensional qualities: system quality, IQ, and SQ. Among these, "system quality" refers to the availability, adaptability, credibility, and effectiveness of the e-commerce system; IQ refers to the quality of the site's content; and SQ is used to evaluate the correlation between the user and the system [17] Similarly, the successful model of the D&M IS has also been extensively discussed and validated with a wide range of applicability. This study has followed D&M IS model among these models because of its wide range of relevancy and competency to identify the satisfaction and intention to use UDC by the citizen.

### 3.1. Research model

**Fig 1:**
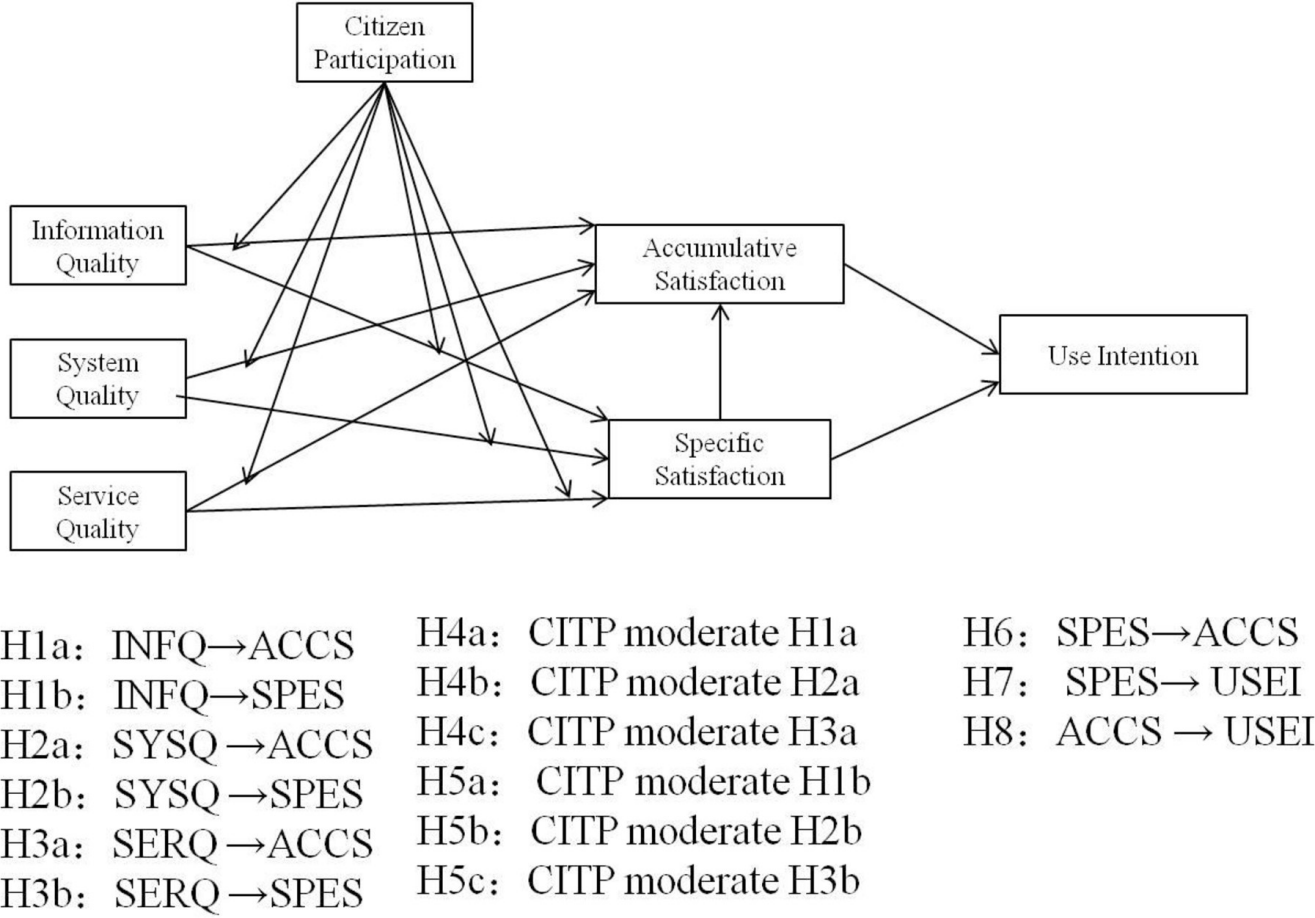
Research model with the hypothesis.

After analyzing the modified D&M IS model, quality of service appraised into three dimensions, i.e. information, service, and system. Zhang (2009) categorized the satisfaction into two types, i.e. a) Specific satisfaction and b) Accumulative satisfaction [18]. Authors also used these types of satisfaction and mentioned to use intention as a dependent variable and citizen participation as a moderator. Fig 1 shows the proposed model with the hypothesis.

If the digital center has enough and accurate information, then the citizen can get proper access to services. As IQ affects citizen satisfaction, so the hypothesis regarding IQ are:

**H1a:** IQ has a significant positive impact on accumulative satisfaction.

**H1b:** IQ has a significant positive impact on specific satisfaction.

System quality denotes the assessment of the e-government system comprising several electronic and network systems. Systematic services run throughout all the processes such as online birth registration, training service, etc. in a systematic way so that recipients of the service can quickly get the benefits. As all of the procedure depends on the regulation of e-government systems so that citizen could give some ratings to the rules and process to identify system quality. Therefore, the proposed hypothesis regarding system quality are:

**H2a:** System quality has a significant positive impact on accumulative satisfaction.

**H2b:** System quality has a significant positive impact on specific satisfaction.

UDC also gives some private service to its citizens. Many government services have also been completed offline by the UDC. The regular electronic system does not deliver some of the services by the entrepreneur of the UDC. The SQ stated here refers to the assessment of the services provided by the UDC. So the services of UDC are bound to affect the final satisfaction evaluation. Thus, the proposed hypothesis regarding SQ are:

**H3a**: SQ has a significant positive impact on accumulative satisfaction.

**H3b:** SQ has a significant positive impact on specific satisfaction.

At the UDCs in Bangladesh, if the citizen can adequately communicate with the staff and actively provide relevant information, it will be easier for them to obtain more relevant and accurate information. Citizen also helps the digital center to give relevant and accurate information to the staff that will make the team more comfortable to understand the real expectations of the citizens. If there is a lack of willingness to interact and not actively cooperate, it is challenging to grasp comprehensive information and necessary precautions. Therefore, different levels of citizen participation are likely to affect the correlation between the quality of service and satisfaction. This study proposes the following hypothesis also:

**H4a:** Citizens’ participation moderates the relationship between Information Quality (IQ) and accumulative satisfaction.

**H4b:** Citizens’ participation moderates the relationship between system quality and accumulative satisfaction.

**H4c:** Citizens’ participation moderates the relationship between service Quality (SQ) and accumulative satisfaction.

**H5a:** Citizens’ participation moderates the relationship between Information Quality (IQ) and specific satisfaction.

**H5b:** Citizens’ participation moderates the relationship between system quality and specific satisfaction.

**H5c:** Citizens’ participation moderates the relationship between service Quality (SQ) and specific satisfaction.

Specific satisfaction refers to the gap that exists between the actual and expected services. On the other hand, accumulative satisfaction defines overall satisfaction since the first access to e-service. Therefore, the proposed hypothesis is:

**H6:** Specific satisfaction has a significant positive impact on accumulative satisfaction.

Customer satisfaction has an important impact on use intention, which can be assumed that citizen could choose any services that they want in the future. The respondents who will face similar needs may come to UDC. This could reflect the effects of satisfaction on use intention. The proposed hypothesis is:

**H7:** Specific satisfaction has a significant positive impact on the use of intention.

**H8:** Accumulative satisfaction has a significant positive impact on the use of intention.

### 3.2. Research design

#### 3.2.1. Variable measurement

The latent variable of this research took on and has been adapted from the former study, which also used a 5-point Likert scale format. The IQ, system quality and SQ were espoused from Wang and Liao [19, 20]. The scale of satisfaction types was espoused that are proposed by Zhang (2009) and Lin et al. (2011) and the scale of citizen participation espoused that are proposed by Zou et al. (2011) [17, 21, 22]. Table 1 shows the various types of identifying variables.

**Table 1:** Identifying variable.

Information quality(INFQ)	INFQ1. Accuracy of website
	INFQ2. Timely information.
	INFQ3. Most current information.
	INFQ4. The necessity of the information
System quality(SYSQ)	SYSQ1. The system returns answers to my requests quickly.
	SYSQ2 The system makes information easy to access.
	SYSQ3. Overall, the whole system in the service center is of very high quality.
Service quality(SERQ)	SERQ1. Staff members show a willingness to help me solve my probles.
	SERQ2. I feel safe in handling my affairs in the service center.
	SERQ3. The staff members show great interest in my situation.
Specific satisfaction(SPES)	SPES1. The last time, I was very satisfied with the service center.
	SPES2. The e-Government system has met my expectations.
	SPES3. I am satisfied with my decision to use my team’s website
Accumulative satisfaction (ACCS)	ACCS1. My experiences in the service center are always very good.
	ACCS2. The service center always meets my expectations.
	ACCS3. Overall, I am always satisfied with the service center
continuance Intention to use	INTU1. Behaviour
	INTU2. Attitude
Citizen Participation (PAR)	PAR1 I will express my feeling to the staff about the service PAR2 I will give suggestions about the UDC to the staff PAR3 I am happy to cooperate with the staff for related matters

### 3.3. Ethics statement

This research is not reviewed by any ethics committee because there have no specific board or committee have in my country based on this types of study. The form of consent obtained in written and data were analyzed anonymously. This research is based on Human participants.

### 3.4. Data collection

A face-to-face strategy was followed for data collection, where a total of 550 questionnaires were distributed, and 515 were returned, and 16 questionnaires were incomplete. After excluding the 16 questionnaires, 499 questionnaires were used for this analysis. There were 499 respondents from 10 unions of 10 upazila under 8 divisions in Bangladesh interviewed for primary data collection. The questionnaire was prepared for data collection from the citizen who got services from UDC. The succeeding Table 2 has shown the demographic information of the citizen, which has been achieved from the study.

**Table 2:** The profile of the respondents (N = 499).

Characteristic	Range	Frequency	Percentage
Gender	Male	274	55%
	Female	225	45%
Age	< = 20	35	7%
	21–30	155	31%
	31–40	128	26%
	41–50	101	20%
	51–60	62	12%
	> = 61	18	4%
Education	No Education	45	9%
	Primary	40	8%
	High school	72	14%
	College	105	21%
	Bachelor	148	30%
	Master's or above	89	18%
Occupation	Teacher	60	12%
	Farmer	53	11%
	Private Employee	54	11%
	Businessman	55	11%
	Housewife	60	12%
	Student	54	11%
	Retired	53	11%
	No job	52	10%
	Others	58	12%
Income BDT. (monthly)	< = 5000	53	11%
	5001–10,000	95	19%
	10,001–20,000	129	26%
	20,001–40,000	92	18%
	> = 40,001	26	5%
	No income	104	21%
**Characteristic**	**Range**	**Frequency**	**Percentage**
Media to know about UDC	People	140	28%
	Advertisement	150	30%
	UDC entrepreneurs	52	10%
	Public Representative/Govt. Official	103	21%
	Website	54	11%
Time duration of using UDC services	This is the first time	35	7%
	<6 months	40	8%
	6–12 months	64	13%
	1–2 years	157	31%
	2–3 years	140	28%
	>3 years	63	13%
Service way	Auto Help	68	14%
	Staff service	431	86%
Coming time to get one service	one time can finish	213	43%
	Need to come several time	286	57%

## 4. Findings

### 4.1. Measurement model

The measurement model was considered for examining internal reliability, discriminant validity, and convergent validity [23] and is exhibited in Table 3.

**Table 3:** Measurement model.

Constructs	Items	Loadings	CR	Cronbach's Alpha	AVE
Information Quality	INFQ1	0.83	0.92	0.88	0.74
	INFQ2	0.90			
	INFQ3	0.88			
	INFQ4	0.84			
System Quality	SYSQ1	0.86	0.91	0.85	0.77
	SYSQ2	0.90			
	SYSQ3	0.88			
Service Quality	SERQ1	0.90	0.93	0.88	0.81
	SERQ2	0.91			
	SERQ3	0.90			
Accumulative Satisfaction	ACCS1	0.91	0.95	0.92	0.86
	ACCS2	0.94			
	ACCS3	0.92			
Specific satisfaction	SPES1	0.91	0.94	0.90	0.84
	SPES2	0.93			
	SPES3	0.91			
use intention	USEI1	0.91	0.94	0.90	0.83
	USEI2	0.91			
	USEI3	0.90			

AVE = Average Variance Extracted; CR = Composite Reliability.

### 4.2. Internal reliability

Cronbach's alpha and composite reliability were helped to evaluate internal reliability, and the level of 0.70 was considered to accept internal consistency as an indicator [24]. At least 0.50 of Average Variance Extracted (AVE) can be assumed to present convergent validity. If the item loading is well above 0.50, then a related assumption can be made [23]. Table 3 exhibits the loadings, Cronbach's alpha, composite reliability, and AVE for this research. The calculated Cronbach's alpha values range from 0.85 to 0.92. If the values are greater than 0.70, then it supports strong internal reliability. Table 3 also illustrates the expected loadings range (0.83 to 0.94) and AVE range (0.74 to 0.86) are also greater than the suggested levels. Therefore, the situations for convergent validity belong to the satisfied level of this study.

### 4.3. Convergent validity

Convergent validity was assessed by AVE and item loadings criteria. The values of AVE and item loadings with at least 0.50 are assessed to meet the convergent validity criteria [25]. It is seen from Table 3 that the calculated AVE range (0.74 to 0.86) is greater than the recommended thresholds. Likewise, Table 3 reveals the calculated items loading ranging from 0.83 to 0.94 are higher than recommended values. As a result, the provisions of the convergent validity of the measurement tools are satisfied in this research.

### 4.4. Discriminant validity

“Discriminant validity measures the degree to which a concept differs from other concepts and is indicated by a measure not correlating very highly with other measures from which it should theoretically differ” [26]. The square-root of the cross-loading matrix measured discriminant validity. The square root of the AVE of a construct ought to be higher than its relationship with other constructs for the acceptable discriminant validity [27]. Table 4 shows the calculated square root of AVE was higher than the resembling relationship confirming the discriminant validity of the data.

**Table 4:** Correlation matrix and square root of the AVE.

	ACCS	INFQ	SERQ	SPES	SYSQ	USEI
ACCS	**0.93**					
INFQ	0.73	**0.86**				
SERQ	0.69	0.69	**0.90**			
SPES	0.79	0.75	0.72	**0.92**		
SYSQ	0.67	0.71	0.72	0.69	**0.88**	
USEI	0.73	0.76	0.69	0.81	0.67	**0.91**

USEI = Use Intention; SPES = Specific Satisfaction; ACCS = Accumulative Satisfaction; INFQ = Information quality; SERQ = Service Quality; SYSQ = System Quality.

### 4.5. Structural model

The structural model has been developed for identifying the correlation among the constructs in the research model. The bootstrap method has been used to validate the hypotheses regarding significance level 0.05 (p<0.05) [28]. Table 5 shows the correlation between independent and dependent variables by analyzing t-statistics and path coefficient. The result shows that INFQ (t = 6.11, β = 0.24, p<0.05) have a significant effect on accumulative satisfaction and INFQ (t = 10.90, β = 0.40, p<0.05) have also tremendous influence on specific satisfaction. It also shows SYSQ (t = 2.38, β = 0.10, p<0.05) have a noteworthy impact on accumulative satisfaction, similarly SYSQ (t = 4.80, β = 0.19, p<0.05) have remarkable dominance on specific satisfaction. In the same way SERQ (t = 3.23, β = 0.13, p<0.05) and SERQ (t = 7.19, β = 0.31, p<0.05) have an important effect on accumulative satisfaction and specific satisfaction. Table 5 also demonstrates SPES (t = 11.70, β = 0.45, p<0.05), ACCS (t = 6.60, β = 0.25, p<0.05) and SPES (t = 16.90, β = 0.61, p<0.05) have an effective impact on use intention of UDC where SPES play a positive role in association with ACCS. Thus, H1a, H1b, H2a, H2b, H3a, H3b, H6, H7, and H8 are supported by the result. According to the result, SPES is a more significant determinant than the ACCS (β = 0.61 versus β = 0.25) in terms of the use intention of UDC in Bangladesh.

**Table 5:** The structural model.

Path	Coefficient (β)	T Statistics	Comments
ACCS -> USEI	0.25	6.60	Supported
INFQ -> ACCS	0.24	6.11	Supported
INFQ -> SPES	0.40	10.90	Supported
SERQ -> ACCS	0.13	3.23	Supported
SERQ -> SPES	0.31	7.19	Supported
SPES -> ACCS	0.45	11.70	Supported
SPES -> USEI	0.61	16.90	Supported
SYSQ -> ACCS	0.10	2.38	Supported
SYSQ -> SPES	0.19	4.80	Supported

R2 for ACCS = 0.683; R2 for SPES = 0.650; R2 for USEI = 0.680; significant at p<0.05.

This model interprets 68% of the variance in accumulative satisfaction to the service of UDC (= 0.683), 65% of the variance in specific satisfaction to the service of UDC (= 0.650), and 68% of the variance in the use intention of UDC (0.680).

### 4.6. Moderating effect of citizen participation

Table 6 illustrates the moderating effects of citizens’ participation. The results exhibit the moderating effects of citizens’ participation between INFQ and ACCS (t = 2.13, β = -0.058, p<0.05), SYSQ and ACCS (t = 2.42, β = -0.076, p<0.05), SYSQ and SPES (t = 2.85, β = -0.083, p<0.05), SERQ and ACCS (t = 2.44, β = -0.059, p<0.05) effects as a moderator. Thus H4a, H4b, H4c, and H5b are supported by the result. On the other hand, we did not find any moderating effect between INFQ and SPES (t = 1.94, β = -0.070, p<0.05) and SERQ and SPES (t = 1.60, β = -0.058, p<0.05). So, H5a and H5c are not supported by this research model. So, based on Table 6, it might be presumed that citizen participation has a tremendous influence on using UDC in Bangladesh.

**Table 6:** The moderating effect of citizen participation.

Path	Moderator	Path Coefficient	T Statistics	Comments
INFQ -> ACCS	CITP	-0.058	2.130	Supported
INFQ -> SPES	CITP	-0.070	1.937	Not supported
SYSQ-> ACCS	CITP	-0.076	2.420	Supported
SYSQ -> SPES	CITP	-0.083	2.851	Supported
SERQ -> ACCS	CITP	-0.059	2.443	Supported
SERQ-> SPES	CITP	-0.058	1.595	Not supported

## 5. Discussion

As an empirical study, this research successfully discovered and tested the influence mechanism linked with several variables that are related to the D&M IS success model. The data obtained from UDC was very important for this empirical study on SQ, satisfaction, and intention to use UDC in Bangladesh. This study mentioned that the data that are used for analysis are adequately acceptable for structural and measurement models. The results indicate that the dimensions of the system quality that SQ, and IQ of the D&M IS model could perform well in the field of e-government.

All of the hypotheses except H5a and H5c passed the level of significance (p<0.05). Both the ACCS and SPES are positively and significantly affected by SYSQ, SERQ, and INFQ. ACCS and SPES also positively influenced USEI with a level of significance (p<0.05). Compared with ACCS, the dominance of SYSQ, SERQ and INFQ on SPES is stronger. Further, the comparison between SPES with USEI and ACCS with USEI, SPES is stronger than the ACCS. One probable explanation is that the investigation of respondents took place just after getting access to services. So, the new conception on INFQ, SYSQ, and SERQ favored them to weigh up SPES with more natural feelings and clear principles. From the perspective of ACCS, only the overall score is provided by the citizen. This score could be subjected exactly and affected a little bit by all of the three dimensions. In addition, it revealed that ACCS is greatly influenced by SPES with the level of significance (p<0.05) and with the path coefficient (0.45) which indicates that the path is stable.

Although there is having the same noteworthy level, the path coefficient of INFQ to SPES is significantly better than those of SERQ and SYSQ. There are two reasons that can explain that the information provided by the e-government has been proved accurate, up to date, and precise that is already known by the citizens. The result also mentioned that SPES posed a more meaningful influence on USEI than ACCS. It can be easily agreed that specific satisfaction contributes more than accumulative satisfaction while using UDC intentionally. To some extent, this study pointed out that the system quality is going through the life cycle from "charisma quality" to "linear quality" to "extrinsic quality" [29]. To reflect all of the quality of service, it was found that SQ, IQ, and system quality have a significant influence on citizen satisfaction and intention to use.

Citizen participation has a significant moderating influence on the correlation between INFQ and ACCS, SYSQ and ACCS, SYSQ, and SPES & SERQ and ACCS. On the other hand, there are no moderating effects on the relationship between INFQ and SPES & SERQ and SPES. From the perspective of the path coefficient, citizen participation plays a moderating role in the relationship between IQ, and accumulative satisfaction, system quality and accumulative satisfaction, system quality and specific satisfaction & SQ and accumulative satisfaction. So, when the high level of citizen participation and proper communication with the staff of digital centers take place, then the effect turns out to be more obvious. The possible reason is that the citizen can get accurate and up to date information through interactive participation by which they can improve their accumulative satisfaction. To understand the functions and features of the system, it attains both of the higher accumulative and specific satisfaction.

Citizen participation has an opposite moderating effect on the path of influence and i.e. when the level of participation is high, IQ, SQ, and system quality increase, the marginal utility level satisfaction especially, the accumulative satisfaction decreases. The possible reason is that though the citizens actively participate and cooperate with the staff, rationally there is a narrow gap exists between the expectation and perception in the context of specific service matter. Citizen participation plays a moderating role in matters related to the quality of service and satisfaction of citizens. So, in this research, citizen participation is related to the customer participation mentioned in past research.

### 5.1. Empirical implication

As a key tool for delivering the services of government, UDCs at the union level in Bangladesh have been playing an essential role in improving the quality of service and satisfaction level of the citizen by engaging common people into various activities. Some practical implications can be generalized from this study.

Firstly, serious attention should be paid to ACCS. Through SPES, it may be considerably and effectively influenced by the SYSQ, INFQ, and SERQ, but the coefficient is negligible and the significance level is not too high. Despite that, all three dimensions of SQ have a very significant effect on ACCS. As discussed earlier, ACCS looks like to playing a facilitating role between SPES and SQ, and also positively and remarkably influences USEI. Hence, it may be concluded that ACCS plays a conclusive role in this model. So, measures need to be carried out in this case simultaneously trying to uplift ACCS.

Secondly, INFQ ought to be considered as an essential element for the enhancement of SPES in comparison to SERQ and SYSQ, where it greatly influenced SPES. The proper, accurate and up to date information of UDC directly influence the evaluation process so that the system quality and SQ should be improved.

Thirdly, some reforms should be carried out so that improvement needed the digital center as well as service providing quality of staff. Comparison between ACCS and SPES has a greater effect on USEI. So, serious attention ought to be paid for improving the overall impression of UDC.

### 5.2. Recommendations

To identify the problems and demands of citizens and ensure the feedback of citizens, UDCs have to focus on essential information sources.

#### 5.2.1. Ensure cooperation of all the dimensions of SQ

Timely, authentic, and latest information should be ensured which are being provided by the UDC, and improvement should be made at the functional and systemic levels of the UDC as well as the development of the capacity of the staff who provides service to the citizen. The study already found that citizens’ satisfaction is affected by the system quality, SQ, and IQ.

#### 5.2.2. Strengthen and standardization of UDC

Based on this research, it is found that UDC has a long vision to serve the common people but it is no longer a new initiative at present. So, the demands and expectations of society are also increasing constantly in terms of getting quality services from UDC. Because of the functioning system of UDC, it is unable to meet the needs of citizens independently. Standardization will effectively enhance the SQ in this case. For upgrading the UDC, the concerned authority should absorb existing research results, strengthen policy learning, and standardization to optimize the public service systematically and promote the continuous improvement of UDC.

#### 5.2.3. To develop the administrative system

In general, it is crucial to upgrade the technical and executive level of administrative systems to improve the quality of information, system, and service.

#### 5.2.4. Encourage citizen to participate in all aspects of service

It is essential to design a good participation situation to enhance the willingness for ensuring citizen’s participation and improve the forms of citizen participation, which give full consideration to citizen’s opinions and ensure a smooth feedback channel. By enhancing citizen participation, UDC can accurately understand the needs of the citizen, optimize and upgrade them in the light of actual conditions so that the entire service process can be followed smoothly and SQ can be upgraded.

## 6. Conclusion

The D&M IS success model of e-government demonstrates three dimensions of SQ which is the important determinant to measure citizen satisfaction. Intention to use the service also depends on citizen satisfaction. This study verified the pertinence of the SQ dimensions of the D&M IS model regarding UDC in Bangladesh and also followed the normative empirical research method that might be effective for further studies. Citizen participation plays a significant role in terms of measuring three dimensions of SQ and also satisfaction. According to the research model, citizens’ accumulative and specific satisfaction depends on all of the system quality, IQ, and SQ. On the other hand, through the system procedures have been fixed but the information and SQ have a tremendous influence on satisfaction. The goal of UDC is to deliver quality services to the citizen in which the utmost efficacious evaluation should come from the citizen so that their participation may invite more attention. This study conducted based on some hypothesis that is tested by using PLS. The study confirmed that system quality, IQ, and SQ play an effective role in citizen satisfaction, and citizen participation also affects both the quality of service and satisfaction except the correlation between INFQ with SPES and SERQ with SPES. The findings of this research will be instrumental for the enhancement of the quality of service of UDC and will help the government to take further policy regarding UDC.

## Supporting information

S1 Data (XLSX)
